# Trans-lumen apposing metal stent rendezvous technique for bile duct stone after the gallbladder drainage

**DOI:** 10.1055/a-2767-1975

**Published:** 2026-04-02

**Authors:** Fumiya Kataoka, Mitsuru Okuno, Tsuyoshi Mukai, Atsushi Tagami, Hiroshi Araki, Hisataka Moriwaki, Masahito Shimizu

**Affiliations:** 1 73505 Department of Gastroenterology, Matsunami General Hospital, Gifu, Japan; 2 First Department of Internal Medicine, Gifu University Hospital, Gifu, Japan

A lumen-apposing metal stent (LAMS) is useful for endoscopic ultrasound-gallbladder drainage (EUS-GBD) in patients who are surgically unfit and with acute cholecystitis (AC 1 ). Here, we report a case of common bile duct (CBD) stone (CBDS) removal using a trans-LAMS rendezvous technique through the EUS-GBD tract.

**Fig. 1:**
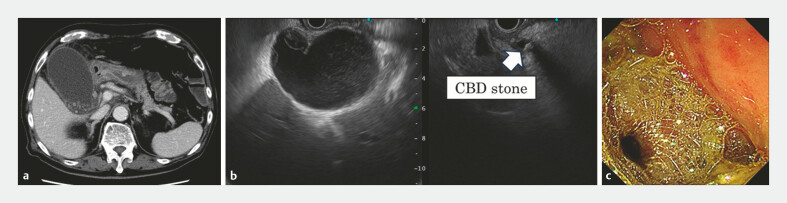
**a** Contrast-enhanced computed tomography (CT) shows a distended gallbladder filled with gallbladder stones. **b** Endoscopic ultrasound (EUS) shows a common bile duct stone (CBDS). **c** A lumen-apposing metal stent was placed transduodenally, resulting in successful gallbladder drainage.

**Fig. 2:**
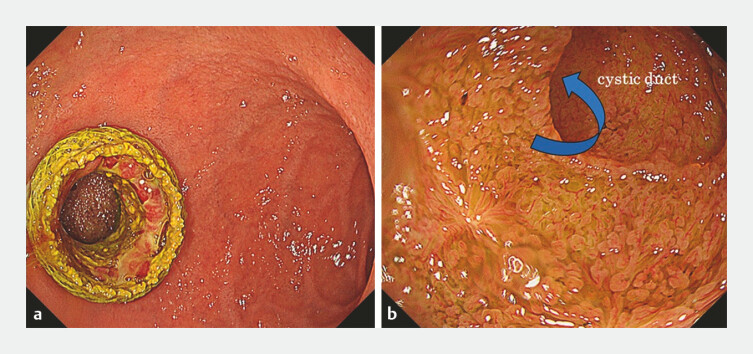
**a** Three months later, the lumen-apposing metal stent (LAMS) remained in the gallbladder and duodenum. **b** The gallbladder was observed via the LAMS tract. No gallbladder stones were detected, and the cystic duct was clearly visible.

**Fig. 3:**
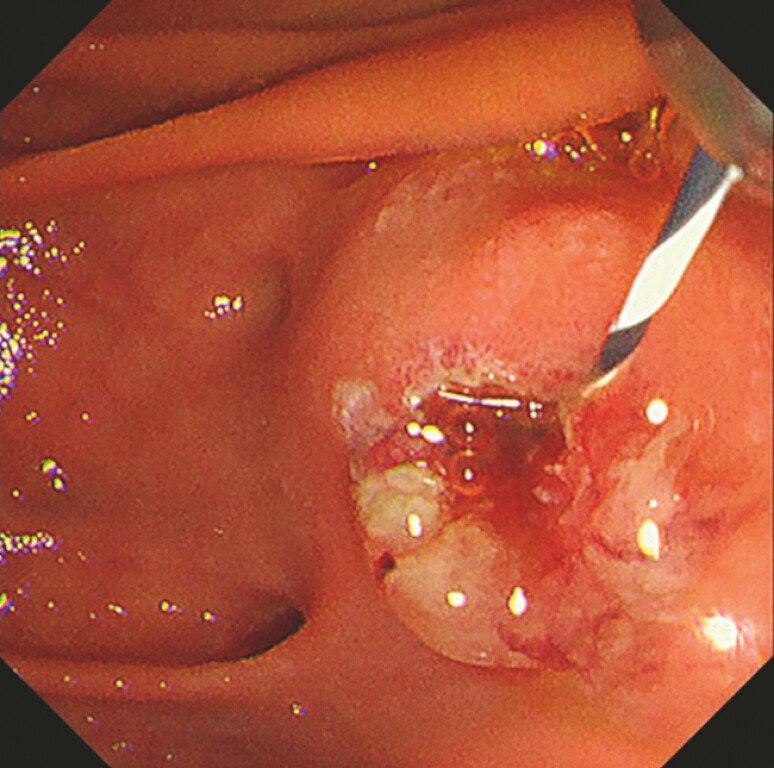
Biliary cannulation was challenging even after endoscopic pancreatic sphincterotomy.

A 92-year-old man presented with fever and abdominal pain. Computed tomography and EUS revealed AC due to gallbladder stones and asymptomatic choledocholithiasis. Cholecystectomy was avoided because of the high risk at an advanced age; instead, we performed EUS-GBD using the LAMS for the AC ( Fig. 1 ) and planned subsequent transpapillary CBDS removal. Three months after the EUS-GBD, an endoscope was inserted into the gallbladder through the LAMS tract, and no gallbladder stones were detected ( Fig. 2 ). Endoscopic retrograde cholangiopancreatography (ERCP) was performed; however, biliary cannulation was challenging, even with the pancreatic guidewire technique and endoscopic pancreatic sphincterotomy. Therefore, the trans-LAMS rendezvous method was performed using an EUS-GBD tract ( Fig. 3 ).

**Fig. 4:**
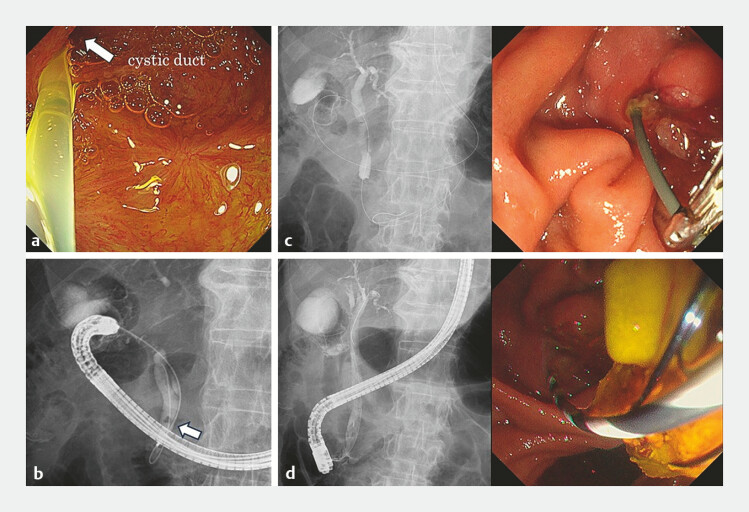
The trans-LAMS rendezvous technique. **a** After the endoscope was advanced into the gallbladder through the LAMS, a 0.025-inch guidewire was passed from the cystic duct into the common bile duct. **b** Cholangiography confirmed choledocholithiasis (white arrow). **c** The guidewire was advanced antegrade into the duodenum via the papilla. **d** Complete stone extraction from the CBD using a retrieval basket. CBD, common bile duct; LAMS, lumen-apposing metal stent.

The trans-LAMS rendezvous technique was performed by passing a 0.025-inch guidewire (EndoSelector; Boston Scientific, Marlborough, MA, USA) from the cystic duct into the CBD using a forward-viewing endoscope (GIF-H290T; Olympus Medical Systems, Tokyo, Japan). The guidewire was then advanced antegradely into the duodenum via the main papilla ( Fig. 4 **a–c** ). We switched to a duodenoscope (TJF-Q290V; Olympus Medical Systems), grasped the guidewire, and successfully cannulated the transpapillary CBD. The CBDS was removed using a retrieval basket after endoscopic sphincterotomy ( Fig. 4 **d** , Video 1 ).

The trans-LAMS rendezvous technique through the EUS-GBD tract for the removal of a CBDS in cases of difficult ERCP cannulation. CBDS, common bile duct stone; ERCP, Endoscopic retrograde cholangiopancreatography; EUS-GBD, endoscopic ultrasound-gallbladder drainage; LAMS, lumen-apposing metal stent.Video 1

This case demonstrated CBDS removal via the trans-LAMS rendezvous technique after a challenging ERCP cannulation procedure. In patients with asymptomatic choledocholithiasis after LAMS placement for EUS-GBD, the trans-LAMS rendezvous technique through the EUS-GBD tract may be an effective option when biliary cannulation during ERCP is challenging.

Endoscopy_UCTN_Code_TTT_1AR_2AH
